# Insights on dramatic radial fluctuations in track formation by energetic ions

**DOI:** 10.1038/srep27196

**Published:** 2016-06-02

**Authors:** Ritesh Sachan, Eva Zarkadoula, Maik Lang, Christina Trautmann, Yanwen Zhang, Matthew F. Chisholm, William J. Weber

**Affiliations:** 1 Materials Science and Technology Division, Oak Ridge National Laboratory, Oak Ridge, Tennessee 37831, USA; 2 Department of Nuclear Engineering, University of Tennessee, Knoxville, TN, 37996, USA; 3 GSI Helmholtzzentrum für Schwerionenforschung GmbH, Planckstrasse, 1, Darmstadt, 64291, Germany; 4 Materialwissenschaft, Technische Universität Darmstadt, Darmstadt, 64287, Germany; 5 Department of Materials Science and Engineering, University of Tennessee, Knoxville, TN, 37996, USA

## Abstract

We report on unexpected dramatic radial variations in ion tracks formed by irradiation with energetic ions (2.3 GeV ^208^Pb) at a constant electronic energy-loss (~42 keV/nm) in pyrochlore-structured Gd_2_TiZrO_7_. Though previous studies have shown track formation and average track diameter measurements in the Gd_2_Ti_x_Zr_(1−x)_O_7_ system, the present work clearly reveals the importance of the recrystallization process in ion track formation in this system, which leads to more morphological complexities in tracks than currently accepted behavior. The ion track profile is usually considered to be diametrically uniform for a constant value of electronic energy-loss. This study reveals the diameter variations to be as large as ~40% within an extremely short incremental track length of ~20 nm. Our molecular dynamics simulations show that these fluctuations in diameter of amorphous core and overall track diameter are attributed to the partial substitution of Ti atoms by Zr atoms, which have a large difference in ionic radii, on the B-site in pyrochlore lattice. This random distribution of Ti and Zr atoms leads to a local competition between amorphous phase formation (favored by Ti atoms) and defect-fluorite phase formation (favored by Zr atoms) during the recrystallization process and finally introduces large radial variations in track morphology.

For many years, the study of ion track formation by energetic ion irradiation in ceramics has been a subject of interest for materials physicists seeking to understand and improve the radiation tolerance of materials, as well as to utilize the process to create functional/electronic materials due to unique microstructure modifications^1^,^2^,^3^. Understanding track formation has broad implications for radiation damage for nuclear fuels and waste immobilization^4^,^5^, age-dating and thermochronology studies of the earth crust^6^, improving the tolerance of electronic devices to cosmic radiation in space^7^ and fabricating complex nanostructures^8^ for applications, such as fast ion conduction in solid oxide fuel cells^9^,^10^. Ion track formation takes place on time scales of 10 to 100 picoseconds, due to inelastic energy loss by energetic ions to electrons and subsequent energy transfer back to the lattice through electron-phonon coupling. The resulting thermal spike causes atomic rearrangement in almost cylindrical channels with nano-sized cross-sections, commonly called ion tracks, along the penetration path of ions in materials.

This work is focused on ion track formation in pyrochlore-structured Gd_2_TiZrO_7_, which is a widely studied material system due to its interesting physical and chemical properties^11^,^12^. Previously, studies have characterized track formation in Gd_2_TiZrO_7_ induced by swift heavy ions (>~50 MeV) and reported on different aspects, such as track structure, morphology and its dependence on electronic energy-loss, based on different characterization techniques, such as X-ray diffraction (XRD), Raman spectroscopy and transmission electron microscopy (TEM)^8^,^13^,^14^,^15^,^16^,^17^,^18^. Among these techniques, XRD and Raman spectroscopy were used to provide macroscopic and averaged information, while TEM results provided atomic scale details^14^,^16^. The effect of electronic energy-loss (as well as selection of incident ion and its energy) on the track morphology has been thoroughly discussed. While it is well established that the structure of an ion track in Gd_2_TiZrO_7_ contains an amorphous core surrounded by a concentric defect-fluorite shell layer, the atomic-level details on the local variability in amorphous core diameter and shell-layer thickness have been elusive.

There are a few issues that need to be addressed in order to understand the structural characteristics of ion tracks beyond present knowledge. For instance, it is generally accepted that ion tracks in various Gd_2_Ti_x_Zr_(1−x)_O_7_ compositions are uniformly cylindrical shaped channels with extremely small variations (±0.4 nm) in overall track diameter over significantly long track lengths (~40 μm) due to the constant electronic energy-loss from incident ions with ~1–2 GeV energy^14^. Most TEM investigations have showed coherent images, where details are dominated by thickness, phase and strain contrast. An interpretation of such images is complicated without image simulations and hinders the understanding of actual ion track morphology. Moreover, previous descriptions of track formation have not included the complexity of the recrystallization process and its effect on track morphology. Consequently, any correlative molecular dynamics (MD) simulations and theoretical modeling of ion track formation due to electronic energy loss of ions also become difficult to validate in the absence of quantitative atomic resolution experimental details^19^.

In the present work, we provide new insights on the role of recrystallization processes on ion track morphology, through the use of correlated atomic resolution incoherent imaging and MD simulations, that advance the existing knowledge of the subject. We performed nanoscale depth-dependent high angle annular dark field (HAADF) plan view imaging of ion tracks in a 5^th^ order aberration corrected scanning transmission electron microscope (STEM), which provided the incoherent images and ability to three-dimensionally reconstruct an ion track to get morphological information with atomic resolution^20^. This method has an advantage over cross-sectional TEM imaging, which is limited to qualitative track morphology information. Furthermore, MD simulations, based on the inelastic thermal spike model and two-temperature approach^2^, were conducted to better understand the recrystallization process of ion track formation in Gd_2_TiZrO_7_. The MD results are quantitatively consistent with the experimental results. This work provides an improved understanding of the complex nature of energetic ion-lattice interactions and recrystallization process in Gd_2_TiZrO_7_ pyrochlore materials by an integrated study of correlative HAADF/STEM and MD simulations.

## Results

**Figure 1:**
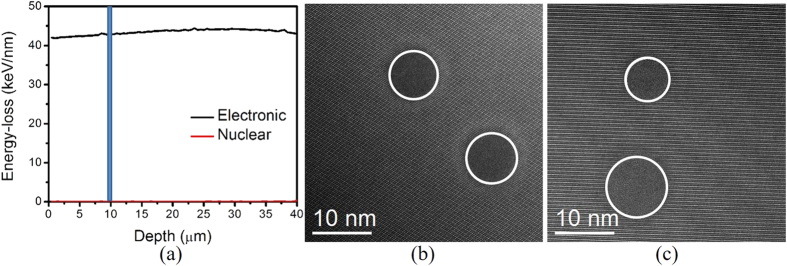
(**a**) Electronic and nuclear energy-loss 2.3 GeV Pb ions as a function of penetration depth in Gd_2_TiZrO_7_. Plan view HAADF images of ion tracks formed by in polycrystalline Gd_2_TiZrO_7_ at a depth of ~10 μm showing (**b**) same and (**c**) different size track formation. A vertical line drawn in figure (**a**) at ~10 μm denotes the depth at which ion track image analysis is performed.

Electronic (S_e_) and nuclear (S_n_) energy loss (dE/dx) of 2.3 GeV ^208^Pb ions in Gd_2_TiZrO_7_ pyrochlore, estimated using Monte Carlo simulations in SRIM-2013 code, are shown as a function of ion penetration depth in Fig. 1. Ion track formation is predicted in the material, since S_e_ energy loss is dominant and well above the threshold for track formation (~8–10 keV/nm) throughout the sample thickness of 40 μm, while the S_n_ energy loss is negligible^21^. It is evident from the figure that S_e_ energy loss remains almost constant at 42 keV/nm with a variation of less than 5% throughout the sample thickness, which suggests the formation of ion tracks with a constant size and morphology at any depth in the material^22^. Surprisingly, the experimental observations by HAADF imaging in STEM show an unexpected large variation in size of different ion tracks at the same depth in Gd_2_TiZrO_7_ (~10 μm), as shown in Fig. 1(b,c). While two ion tracks of the same diameter of 8 nm are seen in one region of sample, as shown in Fig. 1(b), track diameters are significantly different (7.1 nm and 10.2 nm) in the other region.

**Figure 2:**
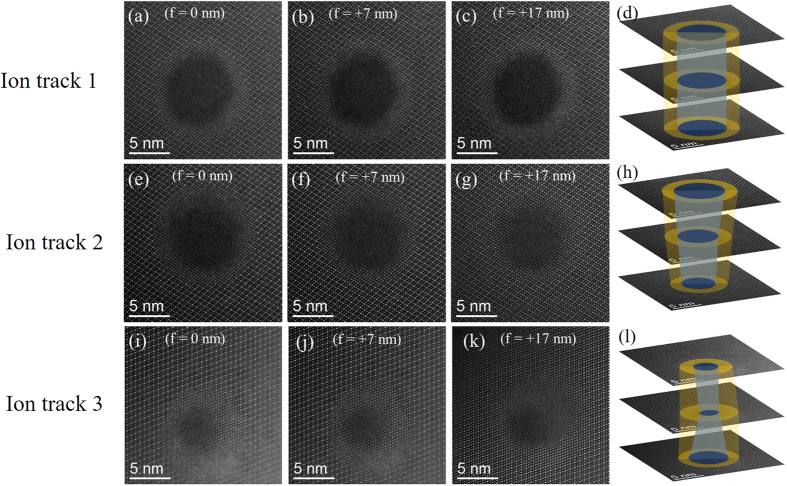
The variation in ion track size and morphology of three different ion tracks within extremely small track length (~20 nm) shown by through-focal series plan-view HAADF images. The through-focal series HAADF images of ion tracks show (**a**–**c**) no variation, (**e**–**g**) decreasing, and (**i**–**k**) abrupt trend change in track diameter. A perspective view of ion track diameter within small depth of ~20 nm is shown in (**d**,**h**,**l**) corresponding to all three ion tracks.

To understand the variation in ion track diameter in different tracks in spite of nearly constant S_e_ energy loss in material, focal series HAADF images were acquired with the electron beam focus changing by 1 nm step into the material. Figure 2 contains HAADF images obtained by focusing at different depths within the specimen, from three different ion tracks in the same sample. The first analyzed ion track is shown in Fig. 2(a–c), where the track diameter is observed to remain unchanged at 7.2 nm throughout the depth of 17 nm. In the second ion track [Fig. 2(e–g)], the track diameter changed from 7.4 nm to 4.5 nm within a track length of 17 nm, which is a change of ~40%. In the third ion track [Fig. 2(e–g)], the diameter changes were also large, similar to the second case (from 2.5 nm to 4.8 nm within a track length of 17 nm). However, it is evident from the HAADF images at *f* = 0, +7 and +17 nm from the surface that this ion track has more radial variations in fractions of amorphous and defect-fluorite phases, unlike the other two cases. A simplified picture of morphology (diameter and length) of all three ion tracks is shown in Fig. 2(d,h,l), illustrating the diametric fluctuations in the ion tracks. The videos of focal series HAADF images acquired by gradually changing the probe focus along the depth in the material are provided in the supplementary section (Video 1, 2 and 3).

**Figure 3:**
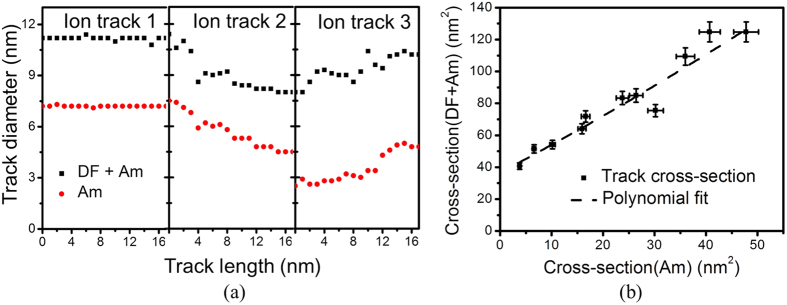
(**a**) Track diameter as a function of track length, based on the depth dependent HAADF analysis. (**b**) Total ion track (Amorphous + Defect fluorite) cross-sectional areal dependency as a function of amorphous area in an ion track. A polynomial is fitted to the data points.

So far, we have focused on the variations in the amorphous core of the ion tracks in Gd_2_TiZrO_7_. In the next part of the work, the emphasis will be on the core/shell morphology of an ion track, where the defect fluorite phase surrounds an amorphous core in the track as a ring. Based on the depth dependent HAADF analysis of three studied tracks, an analysis of total track diameter as a function of track length is shown in Fig. 3(a). The thickness of the defect-fluorite phase layer is 5–6 atomic layers, i.e. ~1.5–2 nm, and remains constant irrespective of the size variations in the amorphous core of the ion track. An exception can be seen in case of ion track 3 in Fig. 2, where the variation of the defect-fluorite phase thickness is due to more dramatic diametric fluctuations of tracks. A plot of total track cross-section (defect fluorite + amorphous) is shown in Fig. 3(b) as a function of amorphous core cross-section. A polynomial fit of the form, 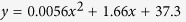, describes the total cross-section (*y*) dependence on the amorphous core cross-section (*x*).

**Figure 4:**
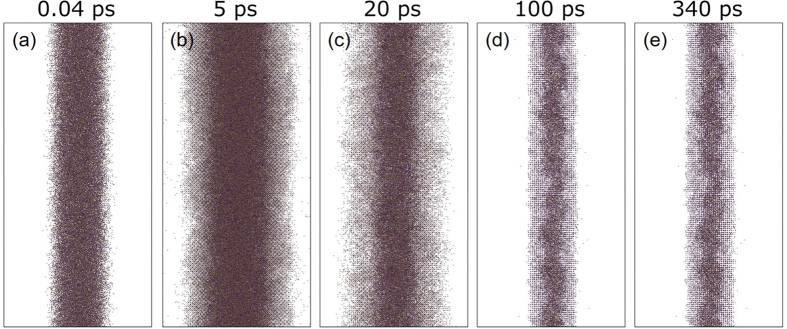
Molecular dynamics simulations of the time evolution of an ion track in Gd_2_TiZrO_7_. For clarity, only the interstitials are shown in the figure, white background represents the ordered atomic structure. The figure illustrates the interstitials formed in Gd_2_TiZrO_7_ after simulation times of (**a**) 0.04, (**b**) 5, (**c**) 20, (**d**) 100 and (**e**) 340 ps. In the intermediate stages of the recrystallization process, interstitials are formed in a larger region than the melt zone of the ion track due to energy transfer to lattice atoms (at 5 and 20 ps). In the final stages of the recrystallization, the diametric variations are evident in the ion track.

**Figure 5:**
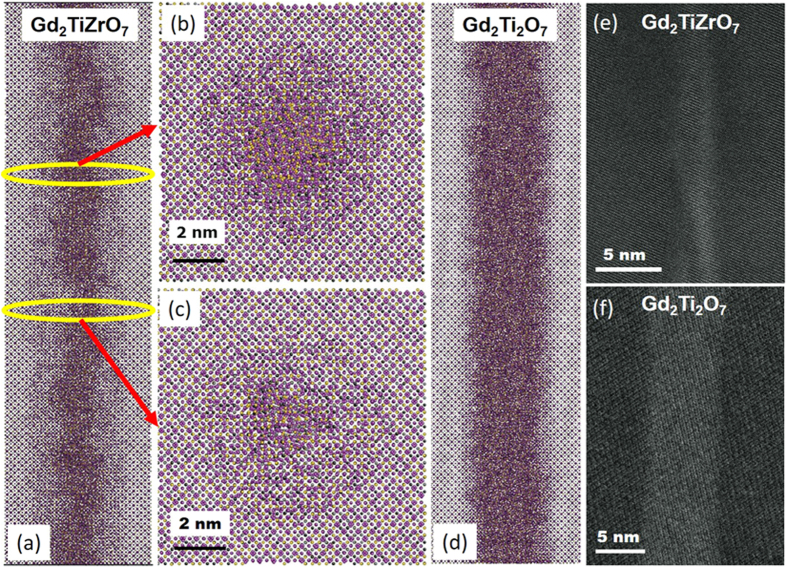
(**a**) A simulated ion track in Gd_2_TiZrO_7_ at 340 ps simulation time, illustrating the variation in the ion track diameter. (**b**,**c**) Cross sections of the ion track at two different depths, indicated in (**a**). The core diameter size is about 4 nm (**b**) and 2 nm (**c**). (**d**) A simulated ion track in Gd_2_Ti_2_O_7_ at 340 ps simulation time, illustrating a uniform diameter ion track. (**e**) A cross-sectional BF image of ion track in Gd_2_TiZrO_7_, (**f**) A cross-sectional BF image of ion track in Gd_2_Ti_2_O_7_.

To understand the evolution of diametric fluctuations in ion track formation in Gd_2_TiZrO_7_, MD simulations as a function of time are performed, as shown in Fig. 4. The figure shows only the interstitials formed during the process for better visual clarity. The initial super-heated melt zone of the thermal spike is shown in Fig. 4(a). This thermal spike induces a shock wave and ejects interstitials into the surrounding matrix, and the melt zone expands into the surrounding matrix, even while the superheated track core cools, as shown in Fig. 4(b). As the molten track core cools further (Fig. 4(c)), interstitials in the ordered pyrochlore matrix begin to relax and epitaxial crystallization of the disordered defect fluorite structure is initiated at the melt-crystalline interface. In the later stages of cooling and crystallization, the defects in the ordered pyrochlore structure mostly recover, the crystallization of the defect fluorite structure ceases, and the remaining molten core quenches to an amorphous structure. This results in an ion track with an amorphous core and defect fluorite shell structure that exhibits significant diametric fluctuations in the core-shell morphology, as shown in Fig. 4(d). The simulations were run for longer time (~340 ps), even after the completion of the track recrystallization, to ensure the stability of track morphology, as shown in Fig. 4(e). From the simulations, it is clear that the observed dramatic diametric variations in the tracks emerge during recrystallization of the molten track containing two very different B-site cations. Figure 5(a) illustrates a slice of recrystallized ion track in Gd_2_TiZrO_7_ after 340 ps of simulation time along the track length, where the variations of the ion track diameter are clearly evident. The ion track consists of an amorphous core, and a defect-fluorite shell structure, in agreement with the experimental findings discussed above. The sectioned images of ion track at two different depths (indicated in Fig. 5(a)) are presented in Fig. 5(b,c). These results clearly illustrate significant change in track diameter over a short increment of track length. To understand the track recrystallization process further, MD simulations were also performed under similar conditions for Gd_2_Ti_2_O_7_ as shown in Fig. 5(d), where the formed track is observed with rather uniform diameter. Likewise, ion tracks in Gd_2_Zr_2_O_7_, which are composed of completely crystallized defect fluorite structure (i.e., no core shell structure), exhibit weak strain contrast and no evidence for significant diametric variation^8^. Bright field (BF) cross-sectional images of the tracks in Gd_2_TiZrO_7_ and Gd_2_Ti_2_O_7_ are shown in Fig. 5(e,f), respectively, which give a projected view of the tracks embedded in the crystalline matrix. The images demonstrate the larger diametric variations for the tracks formed in Gd_2_TiZrO_7_ as compared with tracks in Gd_2_Ti_2_O_7_. Despite the agreement in the core diameter variation, there is some discrepancy between the experimental and the MD results regarding the shell size variation, which in the experiments is not significant. This discrepancy could be attributed to the deficiencies in the interatomic potential, or due to further relaxation processes that occur over the longer time scales of the actual irradiations or the time between irradiation and characterization, and it is not possible to capture such processes in the MD simulations.

## Discussion

**Figure 6:**
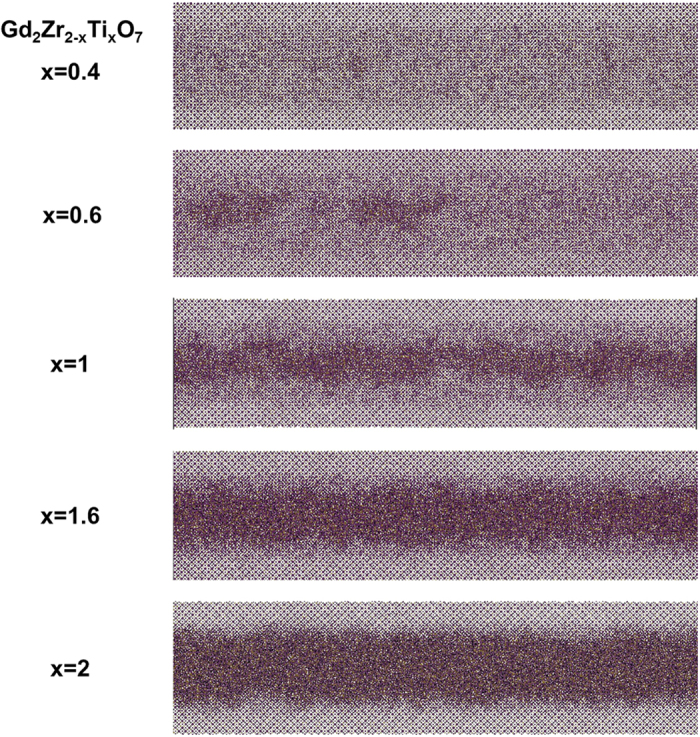
MD simulations of ion track formation in various Gd_2_Zr_(2−x)_Ti_x_O_7_ compositions. Conditions used for simulations are same as described in the methods section. Figure shows the competition between defect fluorite and amorphous phase transformation in the ion track as the effect of changing Zr:Ti ratio on the B atom site.

Since the electronic energy loss by ions in a material is somewhat stochastic in nature, irrespective of the target material, the morphological difference in track formation in Gd_2_TiZrO_7_ and Gd_2_Ti_2_O_7_ is primarily attributed to the random distribution of Ti and Zr atoms on the B-site of the Gd_2_TiZrO_7_ pyrochlore structure. Because of the differences in electron densities and ionic radii for Ti and Zr, local energy dissipation, electron-phonon coupling, and epitaxial crystallization processes are all affected. The ionic radius of Zr is larger than that of Ti (r_Ti_ < r_Zr_) and similar to that of Gd. As a result, the presence of Zr in the pyrochlore structure favors the formation of the defect-fluorite phase during track crystallization due to its low formation energy. On-the-other-hand, the defect fluorite structure is unstable for the small ionic radius of Ti, and Gd_2_Ti_2_O_7_ favors formation of the amorphous phase under track formation conditions. In case of Gd_2_TiZrO_7_, the random distribution of Ti and Zr atoms on the B-site increases the relative stability of the crystallized defect-fluorite structure, which is characterized by the random occupation of the cation sites by Gd, Ti or Zr. This random mixing of Zr with Ti increases the stochastic nature of the local energy dissipation and initiates a competition between defect fluorite and amorphous phase formation^2^,^23^,^24^. This competition between defect fluorite and amorphous phase formation during the crystallization process, which is maximized in the Gd_2_ZrTiO_7_ composition, is believed to be the primary driving force for the dramatic diametric variations in track morphology observed in this material. To provide a better insight, MD simulations for track formation in several Gd_2_Zr_(2−x)_Ti_x_O_7_ compositions (where x = 0.4, 0.6, 1, 1.6 and 2) are shown in Fig. 6 and demonstrate clearly the competition between defect-fluorite and amorphous phase formation as a function of the ratio of Zr and Ti atoms on the B site. Materials with a mix of Ti and Zr show more morphological variations in the formed tracks, while the tracks formed in Gd_2_Ti_2_O_7_ and Gd_2_Zr_2_O_7_ (not shown)^16^ appear more uniform and cylindrical.

In this work, we present a detailed study of dramatic diametric variations in the ion tracks induced by swift heavy ions (2.3 GeV Pb) in Gd_2_TiZrO_7_. Using atomic resolution depth-dependent HAADF imaging of ion tracks, we show the complexity of track formation in Gd_2_TiZrO_7_, where the variation of track diameter is as large as ~40% despite a constant electronic energy loss. By analyzing different ion tracks, it is concluded that these diameter fluctuations are due to the random distribution of Ti and Zr atoms on the B-site of crystal lattice, which locally modifies the energy dissipation and recrystallization processes. In particular, the competition between amorphous phase formation (favored by Ti atoms) and defect-fluorite phase formation (favored by Zr atoms) during the crystallization process induces the observed diametric fluctuations in track morphology.

## Methods

### Ion irradiation

Polycrystalline sample of Gd_2_TiZrO_7_ was prepared by annealing the densified pellets at 800 °C for 24 hrs. The grain size in the samples was ~3–4 μm, which is sufficiently large to negate any effect on bulk track formation^8^,^13^,^25^. Also, the proximity of grain boundaries and the orientation of grains have negligible effects at such high electronic energy-loss^8^,^25^. The samples were further polished to 40 μm thickness. These samples were irradiated at room temperature with 2.3 GeV energy ^208^Pb ions at the beamline X0 of the UNILAC linear accelerator at the GSI Helmholtz Center in Darmstadt, Germany. The ion fluence in the experiment was kept at ~5 × 10^10 ^ions/cm^2^ in order to create isolated ion tracks. The details of sample preparation and ion irradiation are elaborated elsewhere^14^,^18^. The Stopping and Range of Ions in Matter code (SRIM-2013) was used to estimate the nuclear (S_n_) and electronic (S_e_) energy-loss, respectively, as a function of energetic ion penetration depth in Gd_2_TiZrO_7_.

### STEM imaging

The plan-view STEM samples were prepared to analyze the morphology of ion tracks at a depth of ~10 μm from the irradiated surface. The samples for STEM analysis were prepared by mechanically polishing the samples to a thickness of ~10 μm, followed by ion milling in a liquid N_2_ environment^26^. In the mechanical polishing step, the samples were thinned by removing ~25 μm back side and ~5 μm from the irradiated surface. The ion milling was performed using ion guns on both sides of the samples. The samples for cross-sectional imaging were prepared by crushing the irradiated materials into fine powder and subsequently depositing onto a lacey carbon TEM grid. The HAADF and BF imaging was conducted in a 5^th^ order aberration corrected STEM (Nion UltraSTEM 200) operating at 200 kV. The electron probe with 28 pA current was used in the experiment. HAADF images were obtained using a detector with an inner angle of 65 mrad. The BF images were collected with detector having ~15 mrad outer collection angle.

### Molecular Dynamics simulations

For the MD simulations, the DL_POLY MD code was used^27^. The system consists of about 1.2 million atoms and the MD box has 20 nn × 20 nm × 40 nm length. We have used the Minervini and Grimes empirical potentials^28^, joined with the short range ZBL potentials^29^. The ZBL potentials were used for all atomic pairs. The energy deposition from the Pb ions is described by a Gaussian profile with a 2 nm standard deviation^30^. The electron-phonon coupling efficiency is taken to be 0.45. The irradiation of the system was along the z direction under the NVE (constant-volume, constant-energy) ensemble at 300 K, following the equilibration of the system under the NPT (constant pressure, constant-temperature) ensemble. A Langevin thermostat was connected to the atoms contained in a layer of 10 Å along the x and y boundaries of the MD box to emulate the effect of energy dissipation into the sample.

## Additional Information

**How to cite this article**: Sachan, R. *et al*. Insights on dramatic radial fluctuations in track formation by energetic ions. *Sci. Rep*. **6**, 27196; doi: 10.1038/srep27196 (2016).

## Supplementary Material

Supplementary Information

Supplementary Movie S1

Supplementary Movie S2

Supplementary Movie S3
