# Supplementary material for: Insights on dramatic radial fluctuations in track formation by energetic ions
Source: Sci Rep. 2016 Jun 2;6:27196. doi: 10.1038/srep27196 (PMC4890171; doi:10.1038/srep27196)
Supplement: Supplementary Information [file srep27196-s1.pdf]

Supplementary information

Insights on dramatic radial fluctuations in track formation by energetic ions

Ritesh Sachan[1,*], Eva Zarkadoula[1], Maik Lang[2], Christina Trautmann[3,4], Yanwen Zhang[1,5], Matthew F. Chisholm[1], and William J. Weber[5,1]

[1]Materials Science and Technology Division, Oak Ridge National Laboratory, Oak Ridge, Tennessee 37831, USA
[2]Department of Nuclear Engineering, University of Tennessee, Knoxville, TN, 37996, USA
[3]GSI Helmholtzzentrum für Schwerionenforschung GmbH, Planckstrasse, 1, Darmstadt, 64291, Germany
[4]Materialwissenschaft, Technische Universität Darmstadt, Darmstadt, 64287, Germany
[5]Department of Materials Science and Engineering, University of Tennessee, Knoxville, TN, 37996, USA
*sachanr@ornl.gov

Video 1: Sequential depth dependent HAADF images of ion track 1 from the main manuscript. The images were acquired by changing the focus of electron beam with 1 nm step, starting from the surface (represented as f = 0 in the figure 2). Various frames from the video are presented in the manuscript in Fig. 2(a-c).

Video 2: Sequential depth dependent HAADF images of ion track 2 from the main manuscript. The images were acquired by changing the focus of electron beam with 1 nm step, starting from the surface (represented as f = 0 in the figure 2). Various frames from the video are presented in the manuscript in Fig. 2(e-g).

Video 3: Sequential depth dependent HAADF images of ion track 3 from the main manuscript. The images were acquired by changing the focus of electron beam with 1 nm step, starting from the surface (represented as f = 0 in the figure 2). Various frames from the video are presented in the manuscript in Fig. 2(i-k).
